# Biological water quality assessment in the degraded Mutara rangelands, northeastern Rwanda

**DOI:** 10.1007/s10661-019-7226-5

**Published:** 2019-02-08

**Authors:** Marie-Claire Dusabe, Torsten Wronski, Guilherme Gomes-Silva, Martin Plath, Christian Albrecht, Ann Apio

**Affiliations:** 1 Department of Biology, Faculty of Science, Mbarara University of Science and Technology, P. O. Box 1410, Mbarara, Uganda; 2 Department of Wildlife and Aquatic Resources Management, University of Rwanda, Nyagatare Campus, P.O. Box 57, Nyagatare, Rwanda; 3 School of Natural Sciences and Psychology, Liverpool John Moores University, Byrom Street, Liverpool, L3 3AF UK; 4 College of Animal Science and Technology, Northwest A and F University, Yangling, 712100 Shaanxi People’s Republic of China; 5 Department of Animal Ecology and Systematics, Justus Liebig University Giessen, Heinrich-Buff-Ring 26-32, 35392 Giessen, Germany; 6 Jomo Kenyatta University of Agriculture and Technology, Kigali Campus, P.O. Box 3373, Kigali, Rwanda

**Keywords:** Macrozoobenthos, Stream invertebrates, TARISS, Water safety, River pollution, Akagera ecosystem

## Abstract

Rwanda is a heavily overpopulated country that also suffers from overstocking with livestock, especially following the return of war refuges after the civil war (1991–1995). At present, approximately 20% of the human population in Nyagatare District in northeastern Rwanda has no access to clean drinking water and sanitation. We used a biotic index based on the presence of selected families of aquatic macroinvertebrates, derived from the “Tanzania River Scoring System” (TARISS), to assess water quality at *N* = 55 sites in the Mutara grasslands in Nyagatare District. Poor water quality became evident across most sampling sites both in the Muvumba (mean ± SE TARISS score 5.25 ± 0.10) and Karangazi Rivers (4.79 ± 0.12). Using a general linear model, we asked whether direct effects of land use forms and input of anthropogenic wastewater have an impact on water quality. Our results found no immediate effects of both forms of disturbance/pollution, probably because overall water quality was already poor. Our study is intended to serve as a starting point for continuous monitoring of water quality in the Mutara rangelands in NE Rwanda. The method applied here is cost-efficient, requires only basic equipment, and training local students to apply this technique can provide a solid basis for its implementation in future surveys related to public health.

**Electronic supplementary material:**

The online version of this article (10.1007/s10661-019-7226-5) contains supplementary material, which is available to authorized users.

## Introduction

Safe and freely available water is a vital and indispensable resource for humans and their livestock. Clean (drinking) water is important for public health, whether it is used for drinking, domestic use, food production, or to sustain the integrity of agro-ecosystems. Improved water supply and sanitation, and better management of water resources, can increase countries’ economic growth and contribute significantly to poverty reduction (Pearson and McPhedran 2008). Worldwide, 884 million people, i.e., 12% of the global human population, lack a basic drinking-water service (freely available, clean drinking water within a radius of 30 min walk), including 159 million people who are entirely dependent on surface water (WHO 2017). Globally, more than 2 billion people use a drinking water that is contaminated with feces, facilitating the transmission of diseases such as diarrhea, cholera, dysentery, typhus, and polio (WHO 2017).

In Rwanda, about 3.4 million people, i.e., 20% of the total population, have no access to basic drinking water services and more than 5 million people, i.e., 30% of Rwandans, have no toilet (Sekomo et al. 2012). Despite a strong governmental commitment to improve access to clean water, sanitation, and hygiene (Ministry of Infrastructure 2010), surface water in Rwanda is still severely polluted by anthropogenic activities such as the use of fertilizers and pesticides in agriculture (MINIRENA 2011). Furthermore, in Rwanda, settlements and farms are often located along valley slopes where water run-off flushes manure, human excretions, and waste water into rivers and streams, leading to increased levels of dissolved nitrogen. Altogether, these agricultural and domestic activities pollute the surface water and lead to eutrophication and serious public health issues in large parts of Rwanda (Sekomo et al. 2012).

Availability of clean drinking water is often jeopardized during major humanitarian crises like civil wars. Following the civil war and genocide in Rwanda (1990 to 1995), refugees returned from neighboring Uganda and Tanzania with about 700,000 cattle, settled in the Mutara Rangelands and severely overstocked the already oppressed rangelands (Kanyamibwa 1998). Since the end of the 1990s, Rwanda’s economy has grown dramatically, showing stimulating signs of development (Terrill 2012). However, due to still increasing human (483.1 individuals/km^2^; NISR 2014) and livestock densities (64.9 individuals/km^2^; Wronski et al. 2017) in areas like the Mutara rangelands in northeastern Rwanda, surface water is increasingly polluted by anthropogenic activities.

Rwanda is located in the Great Lakes Region of Africa. Its topography gradually rises from the East at an average altitude of 1250 m to the North and West where it culminates in a mountain range called Congo-Nile Divide varying from 2200 to 3000 m altitude. Rwanda possesses a dense hydrographical network, with lakes occupying about 1280 km^2^ and rivers covering 72.6 km^2^. The country is separated into two major catchment areas: the Congo River Basin to the West of the Congo-Nile Divide (covering 33% of the national territory, receiving 10% of all national waters) and the Nile River Basin to the East (covering 67% of the territory, receiving 90% of the national waters; Sekomo et al. 2012). In our study, we aimed to characterize the water quality in the two major affluents to the Akagera River, which represents the main drainage line within the Nile River Basin, using biological water monitoring. We used the Tanzania River Scoring System (TARISS), a biotic index based on the presence of selected families of aquatic macroinvertebrates and their perceived sensitivity to water quality changes (Kaaya et al. 2015). The biological assessment of rivers using aquatic macroinvertebrates is an internationally recognized approach to determine the environmental condition of freshwater bodies (Caspers and Karbe 1967; Baur 1987; Davis and Simon 1994; Knoben et al. 1995; Liess et al. 2008).

Our major aim was to provide an overview of water quality in our study area (i.e., in the Mutara rangelands) and to contrast our findings with assessments that were based on a few physico-chemical parameters only (RNRA 2014), driven by the fact that the surface waters we considered in this study represent a source of drinking water for a large portion of the local human population (app. 30% of Nyagatare District, i.e., about 140,000 persons; NISR 2015). In addition, we also tested for a gradual zonation of macrozoobenthos communities along elevational gradients (Vannote et al. 1980)—important biological background information for the interpretation of data obtained from biological water quality monitoring. We, therefore, assessed geo-physical, physico-chemical and biological characteristics at each sampling site to characterize multivariate, gradual variation in abiotic and biotic conditions in both streams from spring to downstream regions. Moreover, we inspected whether direct effects of land use forms and input of anthropogenic wastewater have a visible impact on water quality. However, one could predict to find no immediate effects, as those factors effect macrozoobenthos communities only downstream, or cumulative effects of several factors related to anthropogenic disturbance/pollution could be more important than single indicators of disturbance/pollution at a given sampling site.

Our study is intended to serve as a starting point for continuous monitoring of water quality in the Mutara rangelands. Biological assessment of water quality is comparatively cost-efficient and requires only basic equipment (Walley and Judd 1993; Czerniawska-Kusza 2005), and training local students by taxonomic experts to apply this technique can provide a solid basis for the implementation of water quality assessments in future surveys related to public health (Nile Basin Initiative 2005).

## Material and methods

### Study area

**Fig. 1:**
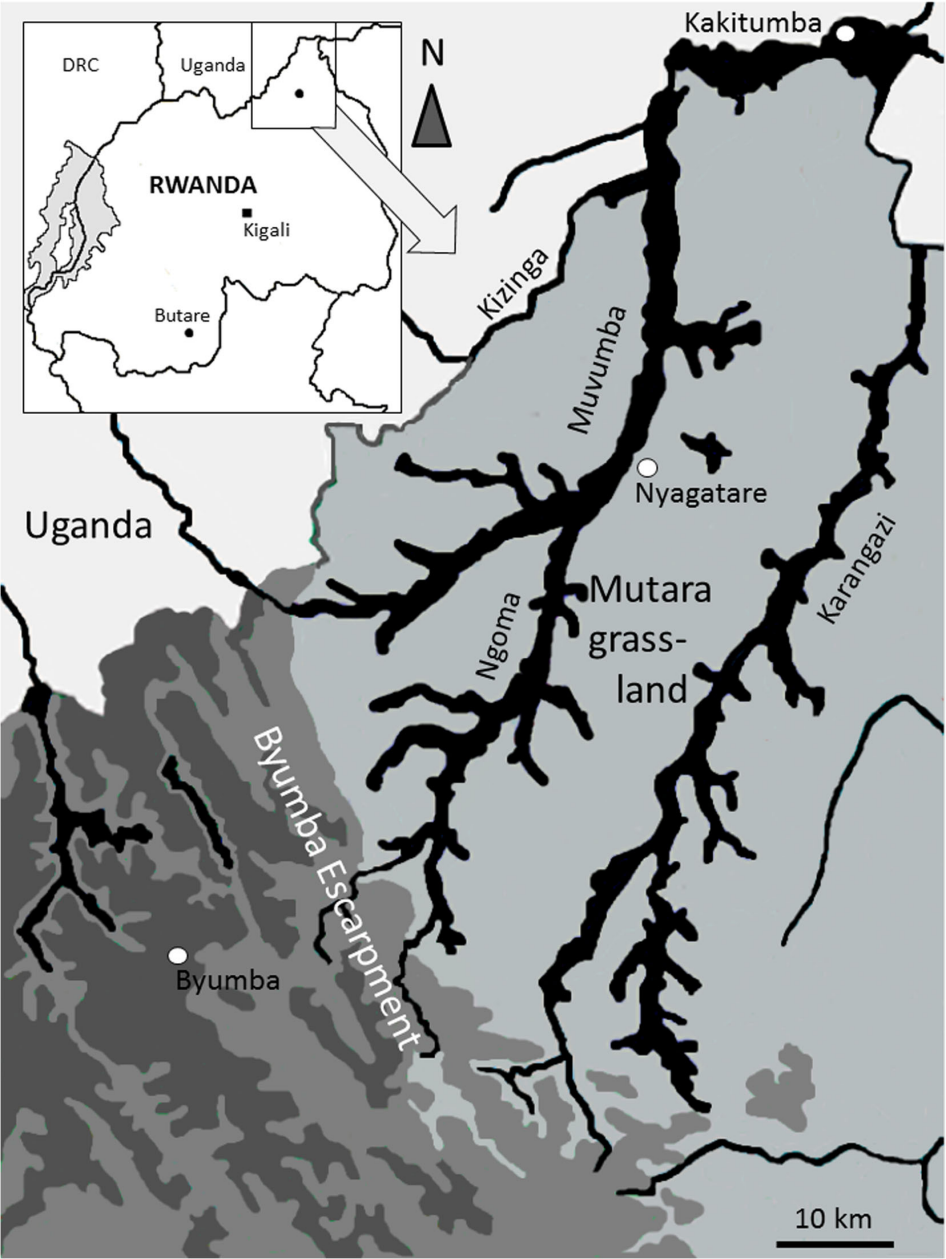
Location of the Mutara grasslands in northeastern Rwanda (inlet) and courses of the Muvumba and Karangazi River systems within the Mutara grasslands. Map modified from Kindt et al. (2014) depicting current land use forms in the study area (gray: degraded grasslands, green/dark grays: escarpment with agriculture, blue/black: flood plains along the two river systems)

We assessed biological water quality in two river systems, i.e., the Muvumba River (including its tributaries Kizinga and Ngoma), and the Karangazi River, both of which are crossing the degraded and overstocked Mutara grasslands in northeastern Rwanda (Fig. 1). Both rivers originate from the Byumba escarpment located to the South and West of the grassland. The escarpment was formed as a result of the uplift of the eastern rift shoulder of the Albertine Rift Valley, comprising a Jurassic to mid-Cretaceous surface ubiquitously covered by alumino-ferruginous laterites (Rossi 1980). The escarpment is deeply dissected into flat-topped sinuous ridges originally vegetated by “Lake Victoria transitional rain forest” (Kindt et al. 2014), abruptly separating the Byumba surface from the much younger, more acidic and granitic Kagera surface, forming the Mutara grasslands to the East (Rossi 1980). After almost complete deforestation of the Byumba escarpment (Kindt et al. 2014), the area is nowadays largely covered by terraced slopes with small gardens and fields for subsistence agriculture (Van de Weghe 1990). The Mutara grasslands form—together with the Karagwe District in Tanzania and the Ankole grasslands in southwestern Uganda—the Akagera Ecosystem. The original vegetation of the Mutara comprised vast open grasslands and savannah woodlands that are nowadays heavily degraded and predominantly used for cattle grazing (Wronski et al. 2015a, 2017). Until 1997, large parts of the Mutara grasslands were protected as part of the Akagera National Park and the Mutara Game Reserve (Van de Weghe 1990). After the Rwandan civil war, the Mutara Game Reserve and large parts of Akagera National Park were degazetted, reducing the protected area from an initial surface area of 2800 km^2^ to about 1120 km^2^ (Williams and Ntayombya 1999).

The total catchment area of the Muvumba river system is 3714 km^2^, whereby 1568 km^2^ are located within Rwandan borders and another 2146 km^2^ in Uganda (WFGR 2017). The Muvumba catchment finds its source in Rwanda, located in the mountainous and humid central northern part of the country at an altitude of 2030 m. After 22.5 km in Uganda, the Muvumba River flows back into Rwandan territory at an altitude of 1460 m. The length of the Muvumba River within Rwanda is approximately 56.3 km. South of Nyagatare, the Ngoma River, and a few kilometers north, the Kizinga River, contribute their flow to the Muvumba River, flowing in a northeasterly direction before joining the Akagera River at Kakitumba in the northeastern corner of Rwanda at an altitude of about 1280 m (WFGR 2017; Fig. 1). Irrigated and agricultural wetlands (mainly for rice production) occupy a significant part of the catchment in the central and northeastern parts. The southern part of the catchment area is dominated by fields and numerous forest plantations. Natural open grassland is fringing the central part of the river along the border with Uganda.

The catchment area of Karangazi River comprises approximately 900 km^2^, entirely located within Rwandan territory. From its numerous sources in the Byumba escarpment, several creeks form the Karangazi River just before it reaches the Mutara grasslands at an altitude of 1395 m. From here, the river flows in northerly direction, forming a broad intermittent river slowly meandering through swamps of papyrus (*Cyperus papyrus* L.), *Vossia cuspidata* (Roxb.) Griff., and *Echinochloa pyramidalis* (L.) P. Beauv (Van de Weghe 1990). During the dry season, the river shrinks to a small creek, drying off over large parts, with water remaining only in pools and upstream of artificial dams. After about 54 km, the Karangazi drains into the Akagera River at an altitude of 1290 m, just north of the modern Akagera National Park.

### Macrozoobenthos sampling

We collected macroinvertebrate samples from 55 locations, 36 along the Muvumba drainage system, including Kizinga and Ngoma tributaries, and 19 along the Karangazi River system. Sampling locations were set at approximately equal distances, i.e., (mostly) 2 to (maximally) 6 km apart. Sites were chosen to reflect the diversity of microhabitats (pools, riffles, runs) and substrate (mud, sand, gravel) available within the stream. To reveal a comprehensive assessment of different habitat types and to avoid an over- or underestimation of the respective organisms/families, samplings were carried out according to the ratio the habitats occupy at the sampling spot.

We obtained benthic macroinvertebrates (larger than 1 mm) using the kick sampling method as well as hand net sampling for leaf litter and sapropel (Hynes 2001). A scoop net (diameter 20 cm, mesh size 1 mm) attached to a long handle was placed on the streambed against the flow, and the substrate in front of the net was agitated by simple kicks and steps. Eventually, we pulled the net against the stream, dredging through the substrate. For leaf litter and other plant debris, we used a handheld kitchen sieve (diameter 20 cm, mesh size 1 mm). Additionally, we removed stones, smaller rocks, and macrophytes from the stream and visually searched for macroinvertebrates. We combined subsamples in a bucket and rinsed the net into the bucket using river water. To standardize sampling efforts, we applied three catches of each collection method at each sampling location (about 5 m of river reaches). Sampling effort was uniform (90 min duration) and was conducted by one person at each location. We separated organisms according to taxonomic groups into screw top plastic containers using a sorting basin and a feather weight forceps. Samples were preserved in 70% ethanol, labeled, enumerated, and brought to the laboratory at the University of Rwanda-Nyagatare Campus.

Macroinvertebrates were determined to family level, since comprehensive keys to species (or genus) level were not available for the region. We recorded the number of families and the number of individuals per family at each sampling site using determination keys developed for southern Africa (Day et al. 1999, 2001a, b, 2002; Day and de Moor 2002; de Moor and Day 2002; de Moor et al. 2003; Stals and de Moor 2007). For Trichoptera, Plecoptera, and Hemiptera, comprehensive keys were not available and, thus, determination relied on Scott (1974, 1976, 1983, 1993), Zwick (2004), and an unpublished key for common families of Hemiptera (Unwin 2001).

### Hydromorphology and habitat parameters

At each sampling site, we assessed water temperature (°C) and oxygen content (DO) using a combined O_2_/thermometer (WINLAB Data Line Oxygen-Meter, Windaus-Labortechnik, Clausthal-Zellerfeld, Germany). We measured conductivity (μS/cm^2^) using a handheld conductivity tester (Data Line Conductivity-Meter, WINLAB), while pH was determined using a Data Line pH-Meter (WINLAB; mean ± SD, as well as minimum and maximum values are presented in Table 1). Environmental variables were measured at three points and averaged for each sampling location. Physico-chemical characteristics were taken at different times of the day, i.e., whenever macrozoobenthos samples were collected.

**Table 1:** Mean, standard deviation (SD), minimum, and maximum of physico-chemical water parameters assessed at 55 sampling sites along two river systems in the Mutara rangelands

	Mean	SD	Max.	Min.
pH	7.32	0.99	10.14	6.18
Temperature (°C)	23.96	1.79	30.00	19.10
Conductivity (μS cm^−1^)	316.60	228.04	1400.00	56.41
Oxygen content (DO [mg L^−1^])	2.34	0.42	2.99	1.36
Water velocity (m/s)	0.30	0.15	0.62	0.02

Several additional environmental variables were estimated: presence or absence of macrophytes, river width, depth, and altitude at the sampling site. Velocity was estimated by timing the flow of buoyant sticks over a 2-m stretch (Gore 1996). Human impact at each location was visually estimated as the shore structure (three levels: natural forest, grassland with pastoralism, agriculture including sylviculture) and as shore pollution (two levels: anthropogenic wastewater influx present, no pollution discernible). To avoid inter-observer bias, all estimates were taken by the same person throughout the study period.

### Data analysis (water quality assessment)

The family level-based “Tanzania River Scoring System” (TARISS; Kaaya et al. 2015) was applied in this study to measure biological water quality using macroinvertebrates as biological indicators. TARISS is based on the “South African Scoring System” (SASS; Chutter 1998) and was modified by adjusting the list of taxa and by assigning sensitivity weightings to three additional families (Ephemerythidae, Dicercomyzidae and Neritidae). Six SASS taxa known to be endemic to South Africa or Madagascar were excluded (Barbarochthonidae, Hydrosalpingidae, and Petrothrincidae), and three additional taxa (see above) were included, resulting in a total of 99 TARISS taxa (Kaaya 2014). Indices at the family level may under- or overestimate water quality more than those based on the species level but are more adequate in terms of cost-efficiency and availability of taxonomic experts (Czerniawska-Kusza 2005). However, the index does not take into account the absolute abundance of each taxon (family), but merely the presence or absence of each family at the sampling site.

To establish the TARISS score, we assigned a score between 1 and 15 (low sensitivity [1–5], moderate [6–10] and high sensitivity [11–15]) to each identified family per site (Kaaya et al. 2015). In case of the mayfly families Baetidae and Hydropsychidae, we used a sliding scale as these families are mostly represented by several species. The sliding scale operates under the assumption that the more species available at a given site, the less disturbed the site is, such that a sensitivity weighting of “4” is given to Baetidae or Hydropsychidae specimens if only one species was caught per site, “6” if two species were caught, and “12” if more than two species were caught (Kaaya et al. 2015). In the few cases where no TARISS score was provided for an identified family, the taxon was either assigned to the lowest score for that order (e.g., Odonata) or it was scored according to where most other families were grouped (e.g., Diptera, Arynchobdellida).

The TARISS score reflects the families’ perceived susceptibility to pollution, which is based on the principle that different aquatic invertebrates have different tolerances to different oxygen levels (Hynes 1960). For example, mayfly and stonefly families require high oxygen concentrations and are intolerant to oxygen consumption during the degradation of organic pollutants (Hynes 1960) and thus score with the highest sensitivity class of “10–15.” The lowest scoring invertebrates are “worms” (e.g., Oligochaeta) which score “1”, as they are much more tolerant to low oxygen conditions. The overall TARISS score for each site was established by summing all scores for each family present at each site. To obtain a less biased average score, the overall TARISS score at each site was divided by the number of taxa (families) at a given site to produce a variable that is independent of sample size. A larger sample is likely to include more families, thus inflating the TARISS score if not standardized (Hawkes 1998). Biological water quality based on averaged TARISS scores was modified from Aquilina (2013) as follows: 12–15 (very good), 9–12 (good), 6–9 (moderate), 4–6 (poor), and 1–4 (very poor).

### Statistical analysis

To reduce random noise, we subjected physico-chemical parameters such as pH, conductivity, and so forth (but not altitude, river width, etc.) to a smoothing procedure along continuous river stretches (e.g., independently for each affluent) as follows: *y*_*i*_′ = (0.05 × *y*_*i* − 2_) + (0.15 × *y*_*i* − 1_) + (0.6 × *y*_*i*_) + (0.15 × *y*_*i* + 1_) + (0.05 × *y*_*i* + 2_). Predictor variables were then subjected to a factor reduction by means of a principal component analysis (PCA), based on a correlation matrix, using the Varimax option. Three PCs were retained that showed Eigenvalues > 1.0 and cumulatively explained 63.01% of the total variance (Table 2).

**Table 2:** Results of a principal component analysis (PCA) on geo-physical, physico-chemical, and biotic predictor variables collected at all 55 sampling sites (63.01% cumulative variance explained)

Variable	PC1	PC2	PC3
Eigenvalue	2.24	1.76	1.68
Percent variance explained	24.86	19.54	18.61
Altitude (m)	*− 0.812*	0.361	0.046
Width (m)	*0.875*	0.235	0.083
Depth (m)	0.448	0.418	− 0.517
Velocity (m s^−1^)	0.229	0.445	*0.633*
Temperature (°C)	0.412	− 0.240	0.563
Oxygen (ppm)	*− 0.613*	− 0.018	− 0.184
pH	0.043	*0.726*	0.203
Conductivity (μS m^−1^)	0.108	*− 0.775*	0.094
Presence of macrophytes	− 0.021	− 0.119	*− 0.773*

Principal components with an Eigenvalue > 1.0 were retained; axis loadings > 0.6 and < −0.6 are in italics

We asked how various ecological predictor variables (condensed as three PCs, see above) and both indicators of human impact (wastewater influx, shore utilization) affect our biological indicator of water quality (i.e., TARISS scores). To this end, we used TARISS scores as the dependent variable in a general linear model (GLM), in which the three PCs were included as covariates and the two variables characterizing human impact as fixed factors. The two river drainages show marked differences in several geo-physical aspects; most importantly, the Karangazi River temporarily falls dry at times, consisting only of stagnant rest pools. We, therefore, coded “river” as another factor. We initially included all two-way interaction terms but removed non-significant terms from the final model if *P* > 0.1 (*F* < 1.262, *P* > 0.269, partial *η*_p_^2^ < 0.036). Effect strengths were calculated as Wilks’ partial eta square (*η*_p_^2^). All statistical analyses were conducted using SPSS version 19.

## Results

### Qualitative description of biological water quality

**Fig. 2:**
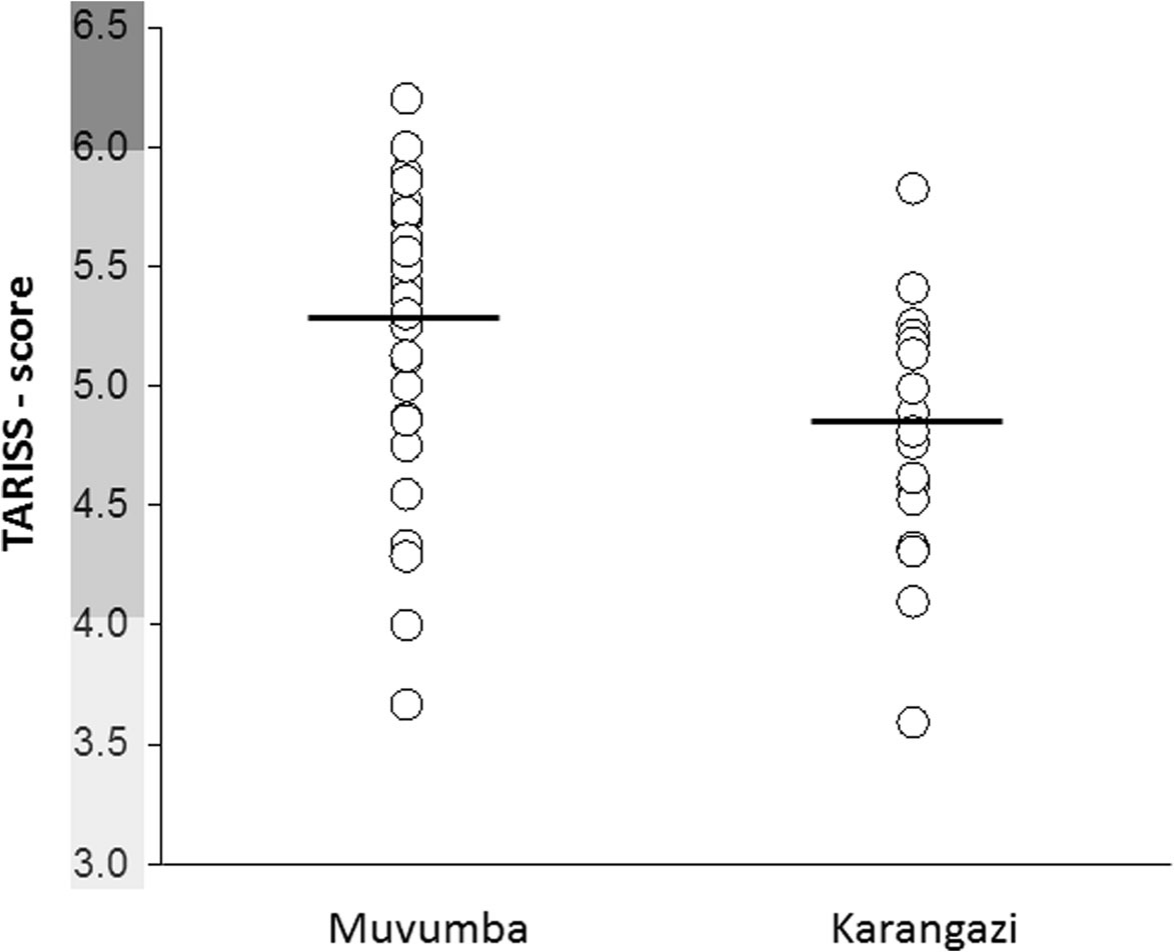
Average TARISS scores for the Muvumba and Karangazi River systems. Water quality classification follows Aquilina (2013), whereby light shading indicates “very poor” quality, moderate shading “poor” quality, and dark shading “moderate” water quality

Following Aquilina (2013), our biological water quality monitoring based on averaged TARISS scores characterized water quality in both river systems as “poor” (i.e., category 4–6), as mean (± SE) scores were determined as 5.25 ± 0.10 for the Muvumba and 4.79 ± 0.12 for the Karangazi River (Fig. 2). Minimum values (Muvumba 3.7, Karangazi 3.6) even reached scores below 4, suggesting “very poor,” i.e., heavily contaminated, biological water quality (1–3; Aquilina 2013). Both sampling sites were situated within the agriculturally used area above or just below the Byumba escarpment. “Moderate” biological water quality (> 6; Aquilina 2013) was measured only at one sampling site, located in the lower course of the Kizinga River, a major affluent to the Muvumba River.

The five most abundant (mean percentage per sampling location ± SE) macrozoobenthos orders encountered in Muvumba and Karangazi Rivers were Odonata (37.8 ± 2.7%, represented by 4 families), followed by Gastropoda (25.5 ± 4.9%; 3 families), Heteroptera (18.5 ± 2.0%; 11 families), Ephemeroptera (17.2 ± 2.0%; 3 families), Diptera (10.2 ± 1.5%; 6 families), and Coleoptera (11.9 ± 1.8%; 19 families). All others, including annelids and hirudineans, decapods, arachnids, bivalves, trichopterans, and plecopterans made up only 9.6 ± 1.5% of the total macroinvertebrates retrieved at each sampling location. Details of macroinvertebrate family abundance and physico-chemical parameters at each sampling locations are presented in Table S1 (Online supplementary material).

### Variation of TARISS scores along both river gradients

PCA on ecological predictor variables (except two indicators of anthropogenic impact/pollution) yielded three PCs. The first PC received high axis loadings from altitude, stream width, and oxygen content (Table 2), characterizing environmental variation ranging from highly oxygenated headwaters to less oxygen-rich downstream sections. The second PC received high axis loadings from pH and conductivity, whereby both variables loaded in opposing directions (Table 2). A decreasing pH with gradually increasing water conductivity is indicative of an influx of acidifying material, decay of organic matter, or presence of acidic soils and base rock along the stream gradients considered in this study. The third PC received high axis loadings from stream velocity and presence of macrophytes, which loaded in opposing directions, reflecting more macrophyte growth in calm stream sections (Table 2).

In our GLM using the TARISS score as the dependent variable, a highly significant effect of the factor river was detected (Table 3). This reflects that mean (± SE) TARISS scores were 5.25 ± 0.10 for the Muvumba River, but somewhat lower (4.79 ± 0.12) in case of the Karangazi River. No significant main effects of both fixed factors related to human impact/pollution were found; likewise, the three PCs capturing environmental variation had no significant main effects. We found a highly significant interaction effect of PC1 × PC2, while the interaction of shore utilization × PC1 only bordered statistical significance (Table 3).

**Table 3:** Results of a GLM using our biological indicator of water quality (TARISS scores) as the dependent variable, three PCs reflecting environmental variation (Table 2) as covariates and two forms of human impact (presence or absence of wastewater influx, shore utilization) as well as “river” (two drainages: Muvumba and Karangazi) as fixed factors

Factor	*df*	*F*	*P*	Wilks’ partial *η*_p_^2^
Shore utilization	2	0.70	0.50	0.03
Pollution	1	0.22	0.64	0.005
River	*1*	*14.21*	*< 0.0001*	*0.24*
PC1	1	0.11	0.74	0.002
PC2	1	1.11	0.30	0.03
PC3	1	0.006	0.94	< 0.0001
PC1 × PC2	*1*	*11.20*	*0.002*	*0.20*
Shore utilization × PC1	2	2.91	0.07	0.12
Error	44			

Significant effects are indicated by italics

**Fig. 3:**
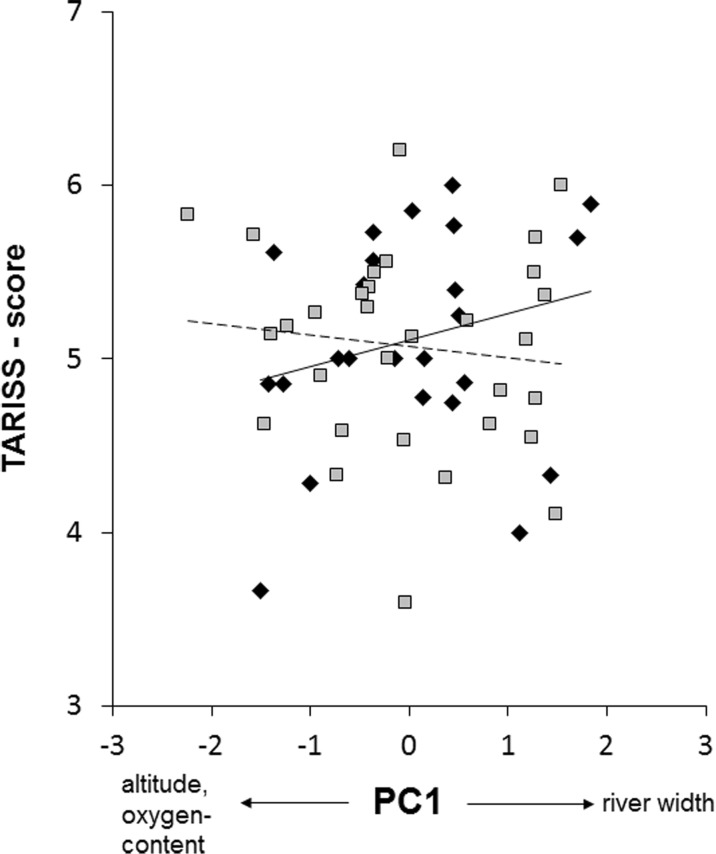
Visualization of the interaction effect of PC1 × PC2 on TARISS scores (see Table 3). Decreasing TARISS scores with increasing values of PC1 (Table 2) become evident for the data with values of PC2 larger than the median (shaded square, dashed line; linear regression: *R*^2^ = 0.014), while slightly increasing TARISS scores with increasing values of PC1 are seen in case of the data with values of PC2 smaller than the median (bold rhomb, solid line; *R*^2^ = 0.056)

To depict the significant interaction effect, we split the data set by the empirical median value of PC2 and show scatterplots and linear regression fits for the dependency of TARISS scores on PC1 for both cohorts of data (Fig. 3). In the cohort of data with values of PC2 smaller than the empirical median value (i.e., relatively lower pH but higher conductivity), we found TARISS scores to increase with increasing values of PC1, i.e., towards river sections in downstream direction. By contrast, in the cohort of data with values of PC2 larger than the empirical median value (relatively higher pH but lower conductivity), no such effect—and even a slight decrease with increasing values of PC1—was observed (Fig. 3). This means that when the influx of acidifying material and/or contact with acidic soils was comparatively strong, increased biodiversity was seen in downstream sections, but this effect did not become apparent when the influx of acidifying material and/or contact with acidic soils was comparatively weak.

## Discussion

### Biological water quality of Muvumba and Karangasi Rivers

The overall biological water quality in both Muvumba and Karangasi Rivers was found to be poor (Fig. 2). Based on assessments of chemical pollution, including measurements of pH, major ions, and fluoride, the water quality of the Muvumba river was announced to have improved from an original evaluation as “strongly modified” in 2009 to “good” in 2014 (RNRA 2014). The discrepancy to our findings may be partly explained by different methodological approaches, as we used macrozoobenthos species to determine water quality. To date, it is widely acknowledged that the results obtained from physico-chemical studies merely reflect the conditions prevailing when the respective sample was taken (Resh et al. 1996). By contrast, biomonitoring methods, i.e., environmental monitoring techniques that utilize living organisms to assess environmental quality, provide an indication of both the current water quality as well as longer-term changes (Resh et al. 1996). Macroinvertebrate communities show a time lag in their recovery after water conditions have improved (Palmer et al. 2010; Leps et al. 2016). This is particularly true for sources of pollution that occur only sporadically (like pesticide influx after heavy rain) but still have a profound effect on biological communities and should be considered when evaluating water quality (Palmer et al. 2010; Leps et al. 2016).

Using scores of biological water quality has become increasingly popular in recent years to investigate the water quality of African rivers. Several studies across the African continent have demonstrated poor water quality based on the TARISS or similar indices (e.g., South Africa: Chutter 1998; Kenya: Ndaruga et al. 2004; Uganda: Kasangaki et al. 2006, 2008; Rwandan rivers draining into Lake Kivu: Wronski et al. 2015b; Tanzania: Kaaya et al. 2015). The index used is based on presence/absence of families. As such, it is less sensitive to seasonality and stochasticity effects compared to approaches based on abundance data. The latter would require significantly higher field and lab efforts and are therefore less practicable at the scale and under conditions of our study. Robustness of indices such as TARISS has been tested and proven repeatedly (Pintoa et al. 2009; Rîşnoveanua et al. 2017). We are, therefore, confident that applying abundance measures would not have changed our overall conclusions.

Domestic waste water (Olomukoro and Ezemonye 2007; Wronski et al. 2015b), agriculture (Ngoye and Machiwa 2004; Mbalassa et al. 2014), and logging of natural forest (Kasangaki et al. 2006, 2008) were identified as the main sources of contamination. The most likely source of organic material in our study system is run-off from cultivated and deforested areas including the influx of human and livestock feces (Bagalwa 2006). However, the two factors we assessed qualitatively in our present study to reflect disturbance and pollution, i.e., shore structure and shore pollution, had no immediate effect on the biological water quality. This may be attributed to the fact that cumulative effects play a more important role than the two factors alone and that effects may manifest themselves only further downstream (Liess and von der Ohe 2005). Moreover, both rivers are generally heavily polluted so that single sources of pollution are unlikely to add much to the overall severe pollution level.

Drinking water polluted by chemicals or fecal bacteria causes severe health problems to humans and their livestock. Pathogenic or disease-causing organisms can affect consumers by causing water-borne diseases such as diarrhea, typhoid fever, or even death if not treated (Yu et al. 2011). The Mutara rangelands largely match the Nyagatare District, which is the largest and second most populated district in Rwanda. In 2012, the district had about 467,000 inhabitants (NISR 2014), of which about 20% had no access to safe water supplies, entirely relying on unprotected water sources such as streams, rivers, cisterns, and poorly constructed wells (Water Aid Rwanda 2016). Rwanda’s commitments to “Vision 2020,” the “Economic Development and Poverty Reduction Strategy (EDPRS),” as well as to the “Millennium Development Goals” have already resulted in good progress in extending water supply and sanitation coverage to the inhabitants of Nyagatare District (AMCOW 2011). This will help to address the pressures related to water pollution, especially where urban runoff drains into rivers and at the same time will help to reduce the risk of water-borne disease epidemics. Moreover, as a response to the water quality and quantity issues described above, Rwanda has established an effective water resources governance framework and committed itself to an “Integrated Water Resources Management (IWRM)” approach (MINIRENA 2011). Regarding sediment as a form of pollution, such rather bureaucratic approaches to combat water pollution often forget to enforce measures that should be taken to regulate agricultural and deforestation activities upriver to avoid further deterioration of downstream conditions (Mbalassa et al. 2014). Heavy rains, in combination with unsustainable agricultural practices, deforestation, and steep slopes contribute to erosion and the consequent siltation of water bodies.

### Variation of TARISS scores along river gradients

The Karangasi River showed somewhat lower diversity of macrozoobenthos communities than the Muvumba River, which reflects that temporal desiccation and formation of isolated pools during the dry season render the Karangasi River uninhabitable for those taxa that do not tolerate phases of hypoxia or complete drying-off of the water, while still providing suitable conditions, e.g., for members of the families Curculionidae, Empididae, Gerridae, Gomphidae, Hydrochidae, Notonemouridae, Scirtidae, and others (Della-Bella et al. 2005). TARISS scores increased towards downstream regions across rivers, but only when influx of acidifying material and/or contact with acidic soil and base-rock was high. In theory, increasing biodiversity from lower- towards mid-order stream sections could—at least in part—be a function of increased availability of particulate organic material (POM; Ward and Stanford 1979). If this was the case, then the cohort of data with PC2 < median value (Fig. 3) would represent sites with more influx of organic material, the decay of which brings about a lower pH. We do, however, find this explanation unlikely, as pollution with organic material is strong throughout our study system. According to the “River Continuum Concept” (e.g., Vannote et al. 1980), however, the mid-order reach of a river (epi-potamal) represents a transition zone between the higher elevation, steeply sloped mountain streams (i.e., low-order stream sections; rhithral) and the lower elevation, shallow-sloped alluvial river reach (high-order sections; potamal). As an area of transition between these two hydromorphic conditions, the mid-order reach represents an ecotone, serving either as the downstream or upstream limit of ecological tolerance for many taxa (Allan and Castillo 2007). Rapid faunal replacement thus occurs over a relatively short stream reach. Because of a certain degree of overlap among taxa associated solely with upstream or downstream hydromorphic conditions, an edge effect usually accounts for the higher biotic diversity observed in this river region (Allan and Castillo 2007).

We argue that the following line of argumentation explains the patterns detected in our present study: Degraded upstream sites (both agricultural and deforested) are responsible for the high load of suspended materials in increased runoff from agricultural fields on the steep-sided slopes, increasing the concentration of fertilizers (Sutherland et al. 2002). Fertilizers such as phosphate and nitrate lead to increased alkalinity of the river water and adversely affect the macroinvertebrate abundance and diversity (in our study, this would be reflected by no increased biodiversity towards downstream sections when PC2 was greater than the empirical median; Fig. 3). Only if surface water is traveling the schistose or metamorphous bedrock areas, like that of the Byumba escarpment, it is characterized by low hardness and alkalinity, i.e., low values of pH (Ndayisaba and Mihale 2015). Moreover, under laterite soils, as found in the Mutara rangelands, availability of phosphate will be reduced through the fixation of phosphate by free anions. This will be further enhanced under acidic conditions, since phosphate will be fixed by free iron and aluminum oxides. In our study, the less disturbed condition (i.e., less phosphate and nitrogen) was reflected by slightly increased biodiversity towards downstream sections when PC2 was smaller than the empirical median (Fig. 3), following the pattern predicted from the River Continuum Concept (Vannote et al. 1980).

Overall, effects of abiotic and biotic components were comparatively weak (see *R*^2^ values in Fig. 3), strengthening our argument that river scoring based on TARISS provides a valuable resource for biological water quality assessment in our study area. In other words, the obtained data mainly provide information about the water quality in a given river stretch and only to a minor extent reflect variation along river gradients. Establishing a specific “Rwandan River Scoring System” should be prioritized in future studies to account for regional differences of aquatic invertebrate faunas across the African continent and to allow an effective regional monitoring. However, even though macrozoobenthos communities might be very specific to East Africa (or Rwanda), it will be imperative to compare and align the new index with other indices established in South Africa, e.g., the South African Scoring System (SASS; Chutter 1998) which was already successfully applied to Rwandan rivers draining into Lake Kivu (Wronski et al. 2015b), as well as in Europe (Caspers and Karbe 1967; Baur 1987; Davis and Simon 1994; Knoben et al. 1995; Liess et al. 2008).

## Conclusions

Biological assessment of water quality in the densely populated and overstocked Mutara rangelands revealed a poorer water quality than previously reported (RNRA 2014). This raises several issues related to public health, as a large number of people in our study area rely on surface water to sustain their livelihoods (NISR 2015). We advise the continuous monitoring of water quality in the streams examined herein (as well as other surface water bodies in Rwanda) using the methodological framework described in our present study. Water protection measures are urgently required, for example, to reduce waste water influx (via sanitation programs) and to maintain soil integrity in upstream areas (e.g., via the implementation of protected areas and banning of agricultural practices in riverine forests) (Kasangaki et al. 2006, 2008).

## Electronic supplementary material

Table S1 (DOCX 103 kb)
