# Supplementary material for: Biological water quality assessment in the degraded Mutara rangelands, northeastern Rwanda
Source: Environ Monit Assess. 2019 Feb 8;191(3):139. doi: 10.1007/s10661-019-7226-5 (PMC6373534; doi:10.1007/s10661-019-7226-5)
Supplement: Supplementary file 1 — (DOCX 103 kb) [file 10661_2019_7226_MOESM1_ESM.docx]

**Online supplementary material**

**Table S1**

Details of macro-invertebrate abundance and physico-chemical parameters (pH, temperature, conductivity, oxygen and velocity) at 55 sampling locations (36 Muvumba, 19 Karangasi) included in this study.

| Station | River | Family | Abundance  (individuals) | pH | Temp. [°C] | Conductivity [μS cm-1] | Oxygen  (DO [mg L-1]) | Velocity  [m/s] |
| --- | --- | --- | --- | --- | --- | --- | --- | --- |
| 2 | Muvumba | Salifidae | 1 | 10.2 | 23.5 | 251.0 | 1.9 | 4 |
|  |  | Lymnaeidae | 1 |  |  |  |  |  |
|  |  | Planorbidae | 20 |  |  |  |  |  |
|  |  | Baetidae | 4 |  |  |  |  |  |
|  |  | Belostomatidae | 1 |  |  |  |  |  |
|  |  | Naucoridae | 4 |  |  |  |  |  |
|  |  | Aspidytidae | 1 |  |  |  |  |  |
| 3 | Muvumba | Lymnaeidae | 27 | 9.5 | 29.7 | 224 | 2.2 | 6 |
|  |  | Planorbidae | 21 |  |  |  |  |  |
|  |  | Baetidae | 4 |  |  |  |  |  |
|  |  | Coenagrionidae | 20 |  |  |  |  |  |
|  |  | Haliplidae | 1 |  |  |  |  |  |
|  |  | Scirtidae | 1 |  |  |  |  |  |
|  |  | Chaoboridae | 3 |  |  |  |  |  |
|  |  | Chironomidae | 6 |  |  |  |  |  |
|  |  | Simuliidae | 10 |  |  |  |  |  |
| 5 | Muvumba | Baetidae | 8 | 9.8 | 26.3 | 276.0 | 2.5 | 6 |
|  |  | Heptageniidae | 5 |  |  |  |  |  |
|  |  | Coenagrionidae | 1 |  |  |  |  |  |
|  |  | Corixidae | 4 |  |  |  |  |  |
|  |  | Naucoridae | 2 |  |  |  |  |  |
|  |  | Aspidytidae | 1 |  |  |  |  |  |
|  |  | Dytiscidae | 2 |  |  |  |  |  |
|  |  | Hydraenidae | 1 |  |  |  |  |  |
|  |  | Hydrometridae | 3 |  |  |  |  |  |
| 6 | Muvumba | Caenidae | 1 | 6.65 | 25.6 | 310 | 2.5 | 6 |
|  |  | Heptageniidae | 3 |  |  |  |  |  |
|  |  | Coenagrionidae | 8 |  |  |  |  |  |
|  |  | Corixidae | 3 |  |  |  |  |  |
|  |  | Naucoridae | 3 |  |  |  |  |  |
|  |  | Chrysomelidae | 1 |  |  |  |  |  |
|  |  | Elmidae | 1 |  |  |  |  |  |
|  |  | Haliplidae | 1 |  |  |  |  |  |
|  |  | Hydraenidae | 1 |  |  |  |  |  |
| 7 | Muvumba | Baetidae | 2 | 6.6 | 26.6 | 307.0 | 2.5 | 6 |
|  |  | Heptageniidae | 4 |  |  |  |  |  |
|  |  | Coenagrionidae | 2 |  |  |  |  |  |
|  |  | Libellulidae | 2 |  |  |  |  |  |
|  |  | Naucoridae | 1 |  |  |  |  |  |
|  |  | Aspidytidae | 3 |  |  |  |  |  |
|  |  | Dytiscidae | 1 |  |  |  |  |  |
|  |  | Haliplidae | 1 |  |  |  |  |  |
|  |  | Hydraenidae | 2 |  |  |  |  |  |
|  |  | Chironomidae | 2 |  |  |  |  |  |
| 8 | Muvumba | Baetidae | 2 | 6.9 | 23.3 | 320.0 | 2.7 | 6 |
|  |  | Heptageniidae | 4 |  |  |  |  |  |
|  |  | Belostomatidae | 2 |  |  |  |  |  |
|  |  | Corixidae | 1 |  |  |  |  |  |
|  |  | Aspidytidae | 3 |  |  |  |  |  |
|  |  | Curculionidae | 2 |  |  |  |  |  |
|  |  | Dytiscidae | 1 |  |  |  |  |  |
|  |  | Elmidae | 1 |  |  |  |  |  |
|  |  | Chironomidae | 1 |  |  |  |  |  |
| 9 | Muvumba | Oligochaeta | 8 | 6.9 | 25.3 | 329.0 | 2.4 | 5 |
|  |  | Polychaeta | 2 |  |  |  |  |  |
|  |  | Atyidae | 1 |  |  |  |  |  |
|  |  | Baetidae | 1 |  |  |  |  |  |
|  |  | Heptageniidae | 2 |  |  |  |  |  |
|  |  | Belostomatidae | 1 |  |  |  |  |  |
|  |  | Naucoridae | 2 |  |  |  |  |  |
|  |  | Aspidytidae | 1 |  |  |  |  |  |
|  |  | Hydrochidae | 8 |  |  |  |  |  |
|  |  | Chironomidae | 3 |  |  |  |  |  |
| 10 | Muvumba | Polychaeta | 1 | 6.6 | 24.6 | 270.0 | 3.1 | 4 |
|  |  | Baetidae | 10 |  |  |  |  |  |
|  |  | Heptageniidae | 3 |  |  |  |  |  |
|  |  | Coenagrionidae | 6 |  |  |  |  |  |
|  |  | Libellulidae | 2 |  |  |  |  |  |
|  |  | Perlidae | 4 |  |  |  |  |  |
|  |  | Belostomatidae | 1 |  |  |  |  |  |
|  |  | Corixidae | 2 |  |  |  |  |  |
|  |  | Naucoridae | 6 |  |  |  |  |  |
|  |  | Dytiscidae | 2 |  |  |  |  |  |
|  |  | Elmidae | 4 |  |  |  |  |  |
|  |  | Hydraenidae | 5 |  |  |  |  |  |
|  |  | Hydrochidae | 1 |  |  |  |  |  |
|  |  | Chironomidae | 1 |  |  |  |  |  |
| 11 | Muvumba | Atyidae | 9 | 7.1 | 23.4 | 340.0 | 3.5 | 6 |
|  |  | Heptageniidae | 4 |  |  |  |  |  |
|  |  | Coenagrionidae | 3 |  |  |  |  |  |
|  |  | Libellulidae | 9 |  |  |  |  |  |
|  |  | Notonemouridae | 1 |  |  |  |  |  |
|  |  | Belostomatidae | 1 |  |  |  |  |  |
|  |  | Naucoridae | 1 |  |  |  |  |  |
|  |  | Empididae | 1 |  |  |  |  |  |
|  |  | Chironomidae | 11 |  |  |  |  |  |
| 12 | Muvumba | Planorbidae | 12 | 7.12 | 25.5 | 358 | 3.7 | 4 |
|  |  | Atyidae | 8 |  |  |  |  |  |
|  |  | Heptageniidae | 5 |  |  |  |  |  |
|  |  | Coenagrionidae | 8 |  |  |  |  |  |
|  |  | Libellulidae | 2 |  |  |  |  |  |
|  |  | Naucoridae | 4 |  |  |  |  |  |
|  |  | Notonectidae | 2 |  |  |  |  |  |
|  |  | Dytiscidae | 1 |  |  |  |  |  |
|  |  | Scirtidae | 2 |  |  |  |  |  |
|  |  | Chironomidae | 6 |  |  |  |  |  |
| 13 | Muvumba | Planorbidae | 5 | 7.2 | 23.8 | 362.0 | 2.5 | 4 |
|  |  | Atyidae | 5 |  |  |  |  |  |
|  |  | Baetidae | 1 |  |  |  |  |  |
|  |  | Coenagrionidae | 16 |  |  |  |  |  |
|  |  | Libellulidae | 2 |  |  |  |  |  |
|  |  | Belostomatidae | 1 |  |  |  |  |  |
|  |  | Naucoridae | 4 |  |  |  |  |  |
|  |  | Nepidae | 1 |  |  |  |  |  |
|  |  | Dytiscidae | 1 |  |  |  |  |  |
|  |  | Haliplidae | 1 |  |  |  |  |  |
|  |  | Simuliidae | 1 |  |  |  |  |  |
| 14 | Muvumba | Potamonautidae | 2 | 6.9 | 20.6 | 253.0 | 2.6 | 4 |
|  |  | Baetidae | 7 |  |  |  |  |  |
|  |  | Caenidae | 1 |  |  |  |  |  |
|  |  | Heptageniidae | 3 |  |  |  |  |  |
|  |  | Perlidae | 8 |  |  |  |  |  |
|  |  | Naucoridae | 2 |  |  |  |  |  |
|  |  | Dytiscidae | 1 |  |  |  |  |  |
|  |  | Elmidae | 2 |  |  |  |  |  |
|  |  | Hydraenidae | 3 |  |  |  |  |  |
|  |  | Chironomidae | 1 |  |  |  |  |  |
| 15 | Muvumba | Sphaeriidae | 1 | 6.7 | 24.1 | 230.0 | 2.9 | 4 |
|  |  | Baetidae | 5 |  |  |  |  |  |
|  |  | Heptageniidae | 3 |  |  |  |  |  |
|  |  | Coenagrionidae | 3 |  |  |  |  |  |
|  |  | Belostomatidae | 4 |  |  |  |  |  |
|  |  | Naucoridae | 4 |  |  |  |  |  |
|  |  | Pleidae | 1 |  |  |  |  |  |
|  |  | Hydrometridae | 1 |  |  |  |  |  |
|  |  | Chironomidae | 3 |  |  |  |  |  |
| 16 | Muvumba | Baetidae | 3 | 6.9 | 24.5 | 217.0 | 3.1 | 3 |
|  |  | Caenidae | 1 |  |  |  |  |  |
|  |  | Naucoridae | 7 |  |  |  |  |  |
|  |  | Gyrinidae | 1 |  |  |  |  |  |
| 17 | Muvumba | Tetragnathidae | 1 | 6.9 | 24.3 | 212.0 | 3.2 | 3 |
|  |  | Baetidae | 9 |  |  |  |  |  |
|  |  | Caenidae | 11 |  |  |  |  |  |
|  |  | Heptageniidae | 1 |  |  |  |  |  |
|  |  | Aeshnidae | 1 |  |  |  |  |  |
|  |  | Coenagrionidae | 25 |  |  |  |  |  |
|  |  | Belostomatidae | 2 |  |  |  |  |  |
|  |  | Naucoridae | 12 |  |  |  |  |  |
|  |  | Pleidae | 1 |  |  |  |  |  |
|  |  | Hydraenidae | 1 |  |  |  |  |  |
|  |  | Hydrometridae | 1 |  |  |  |  |  |
|  |  | Scirtidae | 1 |  |  |  |  |  |
|  |  | Hydropsychidae | 1 |  |  |  |  |  |
| 18 | Muvumba | Baetidae | 7 | 10.5 | 22.7 | 352.0 | 3.4 | 6 |
|  |  | Heptageniidae | 2 |  |  |  |  |  |
|  |  | Coenagrionidae | 3 |  |  |  |  |  |
|  |  | Belostomatidae | 4 |  |  |  |  |  |
|  |  | Naucoridae | 3 |  |  |  |  |  |
| 19 | Muvumba | Lymnaeidae | 1 | 9.2 | 22.3 | 279.0 | 3.1 | 5 |
|  |  | Baetidae | 2 |  |  |  |  |  |
|  |  | Coenagrionidae | 9 |  |  |  |  |  |
|  |  | Belostomatidae | 2 |  |  |  |  |  |
|  |  | Naucoridae | 2 |  |  |  |  |  |
|  |  | Gyrinidae | 1 |  |  |  |  |  |
|  |  | Hydraenidae | 1 |  |  |  |  |  |
|  |  | Noteridae | 1 |  |  |  |  |  |
| 20 | Muvumba | Caenidae | 1 | 6.5 | 25 | 270.0 | 3.2 | 3 |
|  |  | Aeshnidae | 2 |  |  |  |  |  |
|  |  | Coenagrionidae | 5 |  |  |  |  |  |
|  |  | Corixidae | 1 |  |  |  |  |  |
|  |  | Naucoridae | 2 |  |  |  |  |  |
|  |  | Notonectidae | 2 |  |  |  |  |  |
|  |  | Gyrinidae | 1 |  |  |  |  |  |
| 21 | Muvumba | Planorbidae | 1 | 9.5 | 25.4 | 96.0 | 3.1 | 5 |
|  |  | Baetidae | 7 |  |  |  |  |  |
|  |  | Caenidae | 5 |  |  |  |  |  |
|  |  | Aeshnidae | 5 |  |  |  |  |  |
|  |  | Coenagrionidae | 6 |  |  |  |  |  |
|  |  | Curculionidae | 1 |  |  |  |  |  |
|  |  | Hydraenidae | 1 |  |  |  |  |  |
|  |  | Chironomidae | 2 |  |  |  |  |  |
| 22 | Muvumba | Lymnaeidae | 5 | 11.07 | 21.8 | 100.0 | 3.5 | 4 |
|  |  | Planorbidae | 2 |  |  |  |  |  |
|  |  | Atyidae | 10 |  |  |  |  |  |
|  |  | Caenidae | 1 |  |  |  |  |  |
|  |  | Coenagrionidae | 3 |  |  |  |  |  |
|  |  | Gomphidae | 8 |  |  |  |  |  |
|  |  | Gerridae | 1 |  |  |  |  |  |
|  |  | Naucoridae | 1 |  |  |  |  |  |
|  |  | Curculionidae | 1 |  |  |  |  |  |
|  |  | Hydraenidae | 1 |  |  |  |  |  |
| 23 | Muvumba | Caenidae | 3 | 6.5 | 25.4 | 76.0 | 3.4 | 4 |
|  |  | Coenagrionidae | 15 |  |  |  |  |  |
|  |  | Naucoridae | 1 |  |  |  |  |  |
|  |  | Chironomidae | 1 |  |  |  |  |  |
| 24 | Muvumba | Oligochaeta | 1 | 11.3 | 26.2 | 65.0 | 3.2 | 6 |
|  |  | Polychaeta | 1 |  |  |  |  |  |
|  |  | Sphaeriidae | 3 |  |  |  |  |  |
|  |  | Planorbidae | 1 |  |  |  |  |  |
|  |  | Potamonautidae | 1 |  |  |  |  |  |
|  |  | Baetidae | 1 |  |  |  |  |  |
|  |  | Caenidae | 1 |  |  |  |  |  |
|  |  | Coenagrionidae | 11 |  |  |  |  |  |
|  |  | Chironomidae | 3 |  |  |  |  |  |
| 25 | Muvumba | Sphaeriidae | 8 | 9.4 | 22.7 | 53.0 | 3.0 | 4 |
|  |  | Lymnaeidae | 1 |  |  |  |  |  |
|  |  | Potamonautidae | 4 |  |  |  |  |  |
|  |  | Baetidae | 2 |  |  |  |  |  |
|  |  | Caenidae | 2 |  |  |  |  |  |
|  |  | Coenagrionidae | 13 |  |  |  |  |  |
|  |  | Hydroscaphidae | 1 |  |  |  |  |  |
| 26 | Muvumba | Glossiphoniidae | 1 | 5.7 | 22.9 | 63.0 | 3.7 | 4 |
|  |  | Sphaeriidae | 24 |  |  |  |  |  |
|  |  | Planorbidae | 23 |  |  |  |  |  |
|  |  | Potamonautidae | 1 |  |  |  |  |  |
|  |  | Coenagrionidae | 55 |  |  |  |  |  |
|  |  | Naucoridae | 1 |  |  |  |  |  |
|  |  | Chironomidae | 14 |  |  |  |  |  |
| 46 | Muvumba | Aeshnidae | 7 | 6.9 | 23.1 | 120.0 | 3.9 | 30 |
|  |  | Coenagrionidae | 57 |  |  |  |  |  |
|  |  | Libellulidae | 3 |  |  |  |  |  |
|  |  | Belostomatidae | 16 |  |  |  |  |  |
|  |  | Naucoridae | 5 |  |  |  |  |  |
|  |  | Gyrinidae | 1 |  |  |  |  |  |
|  |  | Chironomidae | 13 |  |  |  |  |  |
| 47 | Muvumba | Atyidae | 3 | 7.2 | 23.0 | 123.8 | 3.5 | 12 |
|  |  | Potamonautidae | 1 |  |  |  |  |  |
|  |  | Baetidae | 5 |  |  |  |  |  |
|  |  | Aeshnidae | 2 |  |  |  |  |  |
|  |  | Coenagrionidae | 46 |  |  |  |  |  |
|  |  | Gomphidae | 1 |  |  |  |  |  |
|  |  | Libellulidae | 1 |  |  |  |  |  |
|  |  | Belostomatidae | 15 |  |  |  |  |  |
|  |  | Naucoridae | 26 |  |  |  |  |  |
|  |  | Pleidae | 5 |  |  |  |  |  |
|  |  | Gyrinidae | 4 |  |  |  |  |  |
| 48 | Muvumba | Lymnaeidae | 1 | 6.8 | 24.4 | 147.6 | 3.6 | 9 |
|  |  | Baetidae | 16 |  |  |  |  |  |
|  |  | Caenidae | 8 |  |  |  |  |  |
|  |  | Heptageniidae | 3 |  |  |  |  |  |
|  |  | Coenagrionidae | 57 |  |  |  |  |  |
|  |  | Veliidae | 1 |  |  |  |  |  |
|  |  | Aspidytidae | 1 |  |  |  |  |  |
| 49 | Muvumba | Sphaeriidae | 6 | 7.2 | 23.7 | 113.4 | 3.2 | 13 |
|  |  | Atyidae | 10 |  |  |  |  |  |
|  |  | Potamonautidae | 1 |  |  |  |  |  |
|  |  | Coenagrionidae | 1 |  |  |  |  |  |
|  |  | Libellulidae | 3 |  |  |  |  |  |
|  |  | Chaoboridae | 1 |  |  |  |  |  |
|  |  | Chironomidae | 1 |  |  |  |  |  |
| 56 | Muvumba | Lymnaeidae | 1 | 7.4 | 18.0 | 56.6 | 3.1 | 18 |
|  |  | Baetidae | 5 |  |  |  |  |  |
|  |  | Caenidae | 7 |  |  |  |  |  |
|  |  | Coenagrionidae | 16 |  |  |  |  |  |
|  |  | Libellulidae | 8 |  |  |  |  |  |
|  |  | Gyrinidae | 3 |  |  |  |  |  |
|  |  | Chironomidae | 2 |  |  |  |  |  |
| 57 | Muvumba | Baetidae | 2 | 7.2 | 18.8 | 52.0 | 3.1 | 14 |
|  |  | Caenidae | 3 |  |  |  |  |  |
|  |  | Coenagrionidae | 4 |  |  |  |  |  |
|  |  | Libellulidae | 3 |  |  |  |  |  |
|  |  | Belostomatidae | 2 |  |  |  |  |  |
|  |  | Gyrinidae | 1 |  |  |  |  |  |
| 50 | Muvumba | Atyidae | 30 | 6.8 | 21.4 | 992.0 | 3.2 | 20 |
|  |  | Potamonautidae | 2 |  |  |  |  |  |
|  |  | Baetidae | 20 |  |  |  |  |  |
|  |  | Caenidae | 12 |  |  |  |  |  |
|  |  | Coenagrionidae | 17 |  |  |  |  |  |
| 51 | Muvumba | Sphaeriidae | 15 | 6.9 | 21.8 | 883.0 | 3.1 | 8 |
|  |  | Atyidae | 15 |  |  |  |  |  |
|  |  | Potamonautidae | 2 |  |  |  |  |  |
|  |  | Baetidae | 8 |  |  |  |  |  |
|  |  | Caenidae | 9 |  |  |  |  |  |
|  |  | Heptageniidae | 1 |  |  |  |  |  |
|  |  | Aeshnidae | 4 |  |  |  |  |  |
|  |  | Coenagrionidae | 7 |  |  |  |  |  |
|  |  | Belostomatidae | 1 |  |  |  |  |  |
|  |  | Naucoridae | 1 |  |  |  |  |  |
|  |  | Dytiscidae | 1 |  |  |  |  |  |
|  |  | Gyrinidae | 3 |  |  |  |  |  |
|  |  | Haliplidae | 2 |  |  |  |  |  |
|  |  | Hydrometridae | 1 |  |  |  |  |  |
|  |  | Chironomidae | 2 |  |  |  |  |  |
|  |  | Culicidae | 4 |  |  |  |  |  |
| 52 | Muvumba | Atyidae | 1 | 6.9 | 22.6 | 632.0 | 2.3 | 20 |
|  |  | Potamonautidae | 1 |  |  |  |  |  |
|  |  | Baetidae | 2 |  |  |  |  |  |
|  |  | Coenagrionidae | 7 |  |  |  |  |  |
|  |  | Dytiscidae | 1 |  |  |  |  |  |
|  |  | Haliplidae | 1 |  |  |  |  |  |
|  |  | Hydrometridae | 1 |  |  |  |  |  |
|  |  | Chironomidae | 4 |  |  |  |  |  |
| 53 | Muvumba | Atyidae | 16 | 6.7 | 22.8 | 638.0 | 2.7 | 18 |
|  |  | Baetidae | 4 |  |  |  |  |  |
|  |  | Coenagrionidae | 28 |  |  |  |  |  |
|  |  | Libellulidae | 23 |  |  |  |  |  |
|  |  | Belostomatidae | 2 |  |  |  |  |  |
|  |  | Corixidae | 6 |  |  |  |  |  |
|  |  | Naucoridae | 18 |  |  |  |  |  |
|  |  | Pleidae | 6 |  |  |  |  |  |
|  |  | Aspyditidae | 4 |  |  |  |  |  |
|  |  | Dytiscidae | 3 |  |  |  |  |  |
| 54 | Muvumba | Baetidae | 1 | 7.8 | 24.1 | 635.0 | 3.2 | 12 |
|  |  | Caenidae | 1 |  |  |  |  |  |
|  |  | Heptageniidae | 4 |  |  |  |  |  |
|  |  | Naucoridae | 4 |  |  |  |  |  |
|  |  | Dytiscidae | 8 |  |  |  |  |  |
|  |  | Haliplidae | 4 |  |  |  |  |  |
|  |  | Hydrometridae | 5 |  |  |  |  |  |
|  |  | Chironomidae | 3 |  |  |  |  |  |
|  |  | Culicidae | 7 |  |  |  |  |  |
| 55 | Muvumba | Lymnaeidae | 2 | 7.3 | 23.8 | 472.0 | 2.8 | 14 |
|  |  | Atyidae | 1 |  |  |  |  |  |
|  |  | Potamonautidae | 4 |  |  |  |  |  |
|  |  | Baetidae | 3 |  |  |  |  |  |
|  |  | Caenidae | 2 |  |  |  |  |  |
|  |  | Heptageniidae | 2 |  |  |  |  |  |
|  |  | Aeshnidae | 8 |  |  |  |  |  |
|  |  | Coenagrionidae | 16 |  |  |  |  |  |
|  |  | Gomphidae | 1 |  |  |  |  |  |
|  |  | Libellulidae | 1 |  |  |  |  |  |
|  |  | Belostomatidae | 5 |  |  |  |  |  |
|  |  | Pleidae | 3 |  |  |  |  |  |
|  |  | Aspidytidae | 1 |  |  |  |  |  |
|  |  | Gyrinidae | 3 |  |  |  |  |  |
|  |  | Hydrometridae | 1 |  |  |  |  |  |
|  |  | Chironomidae | 2 |  |  |  |  |  |
| 27 | Karangazi | Hirudinidae | 1 | 6.2 | 25.1 | 311.0 | 2.5 | 12 |
|  |  | Sphaeriidae | 1 |  |  |  |  |  |
|  |  | Ampullariidae | 32 |  |  |  |  |  |
|  |  | Lymnaeidae | 1 |  |  |  |  |  |
|  |  | Planorbidae | 5 |  |  |  |  |  |
|  |  | Baetidae | 1 |  |  |  |  |  |
|  |  | Coenagrionidae | 13 |  |  |  |  |  |
|  |  | Libellulidae | 2 |  |  |  |  |  |
|  |  | Belostomatidae | 2 |  |  |  |  |  |
|  |  | Naucoridae | 2 |  |  |  |  |  |
|  |  | Notonectidae | 2 |  |  |  |  |  |
|  |  | Ochteridae | 1 |  |  |  |  |  |
|  |  | Pleidae | 8 |  |  |  |  |  |
|  |  | Dytiscidae | 1 |  |  |  |  |  |
|  |  | Elmidae | 3 |  |  |  |  |  |
|  |  | Haliplidae | 3 |  |  |  |  |  |
|  |  | Hydraenidae | 1 |  |  |  |  |  |
|  |  | Chironomidae | 3 |  |  |  |  |  |
| 28 | Karangazi | Hirudinidae | 1 | 6.1 | 24.2 | 370.0 | 2.9 | 7 |
|  |  | Sphaeriidae | 4 |  |  |  |  |  |
|  |  | Ampullariidae | 6 |  |  |  |  |  |
|  |  | Baetidae | 2 |  |  |  |  |  |
|  |  | Aeshnidae | 4 |  |  |  |  |  |
|  |  | Perlidae | 1 |  |  |  |  |  |
|  |  | Belostomatidae | 9 |  |  |  |  |  |
|  |  | Corixidae | 3 |  |  |  |  |  |
|  |  | Naucoridae | 18 |  |  |  |  |  |
|  |  | Ochteridae | 1 |  |  |  |  |  |
|  |  | Pleidae | 1 |  |  |  |  |  |
|  |  | Dytiscidae | 1 |  |  |  |  |  |
|  |  | Elmidae | 2 |  |  |  |  |  |
|  |  | Haliplidae | 1 |  |  |  |  |  |
|  |  | Chironomidae | 11 |  |  |  |  |  |
| 29 | Karangazi | Planorbidae | 3 | 6.8 | 21.3 | 357.0 | 3.2 | 0 |
|  |  | Coenagrionidae | 18 |  |  |  |  |  |
|  |  | Libellulidae | 3 |  |  |  |  |  |
|  |  | Belostomatidae | 2 |  |  |  |  |  |
|  |  | Naucoridae | 8 |  |  |  |  |  |
|  |  | Nepidae | 3 |  |  |  |  |  |
|  |  | Pleidae | 1 |  |  |  |  |  |
|  |  | Dryopidae | 1 |  |  |  |  |  |
|  |  | Elmidae | 1 |  |  |  |  |  |
| 30 | Karangazi | Ampullariidae | 1 | 6.5 | 28.1 | 341 | 1.8 | 0 |
|  |  | Lymnaeidae | 1 |  |  |  |  |  |
|  |  | Planorbidae | 1 |  |  |  |  |  |
|  |  | Baetidae | 19 |  |  |  |  |  |
|  |  | Coenagrionidae | 3 |  |  |  |  |  |
|  |  | Libellulidae | 18 |  |  |  |  |  |
|  |  | Belostomatidae | 3 |  |  |  |  |  |
|  |  | Naucoridae | 21 |  |  |  |  |  |
|  |  | Ochteridae | 1 |  |  |  |  |  |
|  |  | Elmidae | 1 |  |  |  |  |  |
|  |  | Haliplidae | 1 |  |  |  |  |  |
|  |  | Hydraenidae | 1 |  |  |  |  |  |
|  |  | Hydrophilidae | 4 |  |  |  |  |  |
|  |  | Chaoboridae | 18 |  |  |  |  |  |
|  |  | Culicidae | 2 |  |  |  |  |  |
| 31 | Karangazi | Salifidae | 1 | 6.6 | 26.3 | 357.0 | 2.4 | 12 |
|  |  | Sphaeriidae | 2 |  |  |  |  |  |
|  |  | Planorbidae | 1 |  |  |  |  |  |
|  |  | Coenagrionidae | 14 |  |  |  |  |  |
|  |  | Libellulidae | 6 |  |  |  |  |  |
|  |  | Nepidae | 1 |  |  |  |  |  |
|  |  | Pleidae | 1 |  |  |  |  |  |
|  |  | Dytiscidae | 12 |  |  |  |  |  |
|  |  | Elmidae | 1 |  |  |  |  |  |
|  |  | Noteridae | 3 |  |  |  |  |  |
|  |  | Sphaeriusidae | 10 |  |  |  |  |  |
| 32 | Karangazi | Hirudinidae | 1 | 6.8 | 24.4 | 392.5 | 1.5 | 40 |
|  |  | Ampullariidae | 11 |  |  |  |  |  |
|  |  | Lymnaeidae | 6 |  |  |  |  |  |
|  |  | Planorbidae | 2 |  |  |  |  |  |
|  |  | Tetragnathidae | 1 |  |  |  |  |  |
|  |  | Baetidae | 7 |  |  |  |  |  |
|  |  | Coenagrionidae | 4 |  |  |  |  |  |
|  |  | Libellulidae | 14 |  |  |  |  |  |
|  |  | Belostomatidae | 6 |  |  |  |  |  |
|  |  | Naucoridae | 14 |  |  |  |  |  |
|  |  | Nepidae | 1 |  |  |  |  |  |
|  |  | Pleidae | 5 |  |  |  |  |  |
|  |  | Haliplidae | 11 |  |  |  |  |  |
|  |  | Noteridae | 1 |  |  |  |  |  |
|  |  | Sphaeriusidae | 1 |  |  |  |  |  |
|  |  | Chaoboridae | 1 |  |  |  |  |  |
|  |  | Chironomidae | 1 |  |  |  |  |  |
| 33 | Karangazi | Sphaeriidae | 16 | 7.1 | 24.7 | 329.9 | 2.5 | 4 |
|  |  | Lymnaeidae | 3 |  |  |  |  |  |
|  |  | Planorbidae | 133 |  |  |  |  |  |
|  |  | Coenagrionidae | 5 |  |  |  |  |  |
|  |  | Libellulidae | 8 |  |  |  |  |  |
|  |  | Gyrinidae | 4 |  |  |  |  |  |
| 34 | Karangazi | Ampullariidae | 1 | 6.9 | 29.0 | 350.0 | 3.1 | 0 |
|  |  | Lymnaeidae | 2 |  |  |  |  |  |
|  |  | Planorbidae | 2 |  |  |  |  |  |
|  |  | Baetidae | 1 |  |  |  |  |  |
|  |  | Coenagrionidae | 2 |  |  |  |  |  |
|  |  | Libellulidae | 18 |  |  |  |  |  |
|  |  | Belostomatidae | 1 |  |  |  |  |  |
|  |  | Naucoridae | 5 |  |  |  |  |  |
|  |  | Dytiscidae | 1 |  |  |  |  |  |
|  |  | Elmidae | 1 |  |  |  |  |  |
|  |  | Helodidae | 1 |  |  |  |  |  |
|  |  | Hydraenidae | 1 |  |  |  |  |  |
|  |  | Noteridae | 1 |  |  |  |  |  |
| 35 | Karangazi | Ampullariidae | 4 | 7.3 | 21.9 | 285.6 | 3.3 | 8 |
|  |  | Planorbidae | 3 |  |  |  |  |  |
|  |  | Tetragnathidae | 1 |  |  |  |  |  |
|  |  | Caenidae | 1 |  |  |  |  |  |
|  |  | Coenagrionidae | 30 |  |  |  |  |  |
|  |  | Libellulidae | 4 |  |  |  |  |  |
|  |  | Belostomatidae | 2 |  |  |  |  |  |
|  |  | Naucoridae | 9 |  |  |  |  |  |
|  |  | Nepidae | 2 |  |  |  |  |  |
|  |  | Gyrinidae | 3 |  |  |  |  |  |
|  |  | Torridincolidae | 1 |  |  |  |  |  |
| 36 | Karangazi | Oligochaeta | 1 | 7.3 | 22.6 | 295.5 | 2.6 | 5 |
|  |  | Baetidae | 7 |  |  |  |  |  |
|  |  | Caenidae | 4 |  |  |  |  |  |
|  |  | Coenagrionidae | 28 |  |  |  |  |  |
|  |  | Libellulidae | 11 |  |  |  |  |  |
|  |  | Belostomatidae | 6 |  |  |  |  |  |
|  |  | Naucoridae | 2 |  |  |  |  |  |
|  |  | Pleidae | 3 |  |  |  |  |  |
|  |  | Chironomidae | 1 |  |  |  |  |  |
| 37 | Karangazi | Hirudinidae | 2 | 7.6 | 26.9 | 229.5 | 2.9 | 18 |
|  |  | Ampullariidae | 1 |  |  |  |  |  |
|  |  | Lymnaeidae | 1 |  |  |  |  |  |
|  |  | Planorbidae | 3 |  |  |  |  |  |
|  |  | Baetidae | 1 |  |  |  |  |  |
|  |  | Caenidae | 3 |  |  |  |  |  |
|  |  | Coenagrionidae | 7 |  |  |  |  |  |
|  |  | Libellulidae | 36 |  |  |  |  |  |
|  |  | Naucoridae | 1 |  |  |  |  |  |
|  |  | Nepidae | 1 |  |  |  |  |  |
|  |  | Pleidae | 1 |  |  |  |  |  |
|  |  | Gyrinidae | 5 |  |  |  |  |  |
|  |  | Haliplidae | 1 |  |  |  |  |  |
|  |  | Helodidae | 1 |  |  |  |  |  |
|  |  | Chironomidae | 3 |  |  |  |  |  |
|  |  | Tipulidae | 2 |  |  |  |  |  |
|  |  | Hydropsychidae | 1 |  |  |  |  |  |
| 38 | Karangazi | Planorbidae | 46 | 6.4 | 22.8 | 188.4 | 2.5 | 4 |
|  |  | Caenidae | 3 |  |  |  |  |  |
|  |  | Coenagrionidae | 7 |  |  |  |  |  |
|  |  | Libellulidae | 35 |  |  |  |  |  |
|  |  | Gyrinidae | 1 |  |  |  |  |  |
|  |  | Hydropsychidae | 1 |  |  |  |  |  |
| 39 | Karangazi | Planorbidae | 2 | 6.6 | 30.0 | 1400.0 | 3.2 | 4 |
|  |  | Baetidae | 20 |  |  |  |  |  |
|  |  | Coenagrionidae | 13 |  |  |  |  |  |
|  |  | Libellulidae | 38 |  |  |  |  |  |
|  |  | Nepidae | 6 |  |  |  |  |  |
|  |  | Notonectidae | 1 |  |  |  |  |  |
|  |  | Pleidae | 1 |  |  |  |  |  |
|  |  | Chaoboridae | 3 |  |  |  |  |  |
|  |  | Chironomidae | 16 |  |  |  |  |  |
|  |  | Culicidae | 1 |  |  |  |  |  |
| 40 | Karangazi | Sphaeriidae | 1 | 6.4 | 21.0 | 370.3 | 2.7 | 0 |
|  |  | Ampullariidae | 5 |  |  |  |  |  |
|  |  | Lymnaeidae | 29 |  |  |  |  |  |
|  |  | Planorbidae | 39 |  |  |  |  |  |
|  |  | Belostomatidae | 2 |  |  |  |  |  |
|  |  | Naucoridae | 5 |  |  |  |  |  |
|  |  | Nepidae | 2 |  |  |  |  |  |
|  |  | Pleidae | 1 |  |  |  |  |  |
|  |  | Dytiscidae | 5 |  |  |  |  |  |
|  |  | Haliplidae | 4 |  |  |  |  |  |
|  |  | Hydraenidae | 1 |  |  |  |  |  |
|  |  | Sphaeriusidae | 5 |  |  |  |  |  |
| 41 | Karangazi | Ampullariidae | 1 | 6.4 | 21.9 | 318.6 | 2.4 | 0 |
|  |  | Lymnaeidae | 2 |  |  |  |  |  |
|  |  | Planorbidae | 11 |  |  |  |  |  |
|  |  | Baetidae | 1 |  |  |  |  |  |
|  |  | Aspidytidae | 1 |  |  |  |  |  |
| 42 | Karangazi | Lymnaeidae | 1 | 6.7 | 23.8 | 325.5 | 2.5 | 6 |
|  |  | Planorbidae | 1 |  |  |  |  |  |
|  |  | Baetidae | 6 |  |  |  |  |  |
|  |  | Caenidae | 4 |  |  |  |  |  |
|  |  | Heptageniidae | 1 |  |  |  |  |  |
|  |  | Coenagrionidae | 72 |  |  |  |  |  |
|  |  | Libellulidae | 7 |  |  |  |  |  |
|  |  | Belostomatidae | 2 |  |  |  |  |  |
|  |  | Naucoridae | 2 |  |  |  |  |  |
|  |  | Nepidae | 1 |  |  |  |  |  |
|  |  | Pleidae | 2 |  |  |  |  |  |
|  |  | Gyrinidae | 2 |  |  |  |  |  |
|  |  | Chironomidae | 1 |  |  |  |  |  |
|  |  | Simuliidae | 1 |  |  |  |  |  |
|  |  | Hydropsychidae | 2 |  |  |  |  |  |
| 43 | Karangazi | Hirudinidae | 2 | 6.5 | 25.4 | 277.2 | 3.1 | 18 |
|  |  | Lymnaeidae | 18 |  |  |  |  |  |
|  |  | Planorbidae | 23 |  |  |  |  |  |
|  |  | Tetragnathidae | 3 |  |  |  |  |  |
|  |  | Baetidae | 1 |  |  |  |  |  |
|  |  | Caenidae | 2 |  |  |  |  |  |
|  |  | Aeshnidae | 1 |  |  |  |  |  |
|  |  | Coenagrionidae | 24 |  |  |  |  |  |
|  |  | Libellulidae | 24 |  |  |  |  |  |
|  |  | Corixidae | 2 |  |  |  |  |  |
|  |  | Naucoridae | 3 |  |  |  |  |  |
|  |  | Nepidae | 1 |  |  |  |  |  |
|  |  | Ochteridae | 1 |  |  |  |  |  |
|  |  | Pleidae | 15 |  |  |  |  |  |
|  |  | Gyrinidae | 10 |  |  |  |  |  |
|  |  | Chironomidae | 1 |  |  |  |  |  |
| 44 | Karangazi | Hirudinidae | 1 | 6.7 | 21.0 | 289.4 | 3.4 | 40 |
|  |  | Salifidae | 1 |  |  |  |  |  |
|  |  | Lymnaeidae | 1 |  |  |  |  |  |
|  |  | Planorbidae | 29 |  |  |  |  |  |
|  |  | Coenagrionidae | 11 |  |  |  |  |  |
|  |  | Libellulidae | 9 |  |  |  |  |  |
|  |  | Pleidae | 5 |  |  |  |  |  |
|  |  | Gyrinidae | 8 |  |  |  |  |  |
| 45 | Karangazi | Lymnaeidae | 42 | 6.0 | 24.4 | 200.0 | 3.1 | 10 |
|  |  | Planorbidae | 6 |  |  |  |  |  |
|  |  | Coenagrionidae | 12 |  |  |  |  |  |
|  |  | Libellulidae | 13 |  |  |  |  |  |
|  |  | Nepidae | 7 |  |  |  |  |  |
|  |  | Pleidae | 4 |  |  |  |  |  |
|  |  | Simuliidae | 2 |  |  |  |  |  |
